# Comparing EUS-guided lumen-apposing metal stents with and without integrated electrocautery for endoscopic drainage of walled-off pancreatic necrosis

**DOI:** 10.1097/eus.0000000000000131

**Published:** 2025-06-27

**Authors:** Barbara Braden, Emmanuel Selvaraj, Christoph F. Dietrich, Noor Bekkali

**Affiliations:** 1 Translational Gastroenterology Unit, Oxford University Hospitals NHS Foundation Trust, Oxford, United Kingdom; 2 Medical Department B, University of Münster, Germany; 3 South Warwickshire University Foundation Trust, Warwick, United Kingdom; 4 Department Allgemeine Innere Medizin, Kliniken Hirslanden, Beau Site, Salem und Permanence, Bern, Switzerland.

**Keywords:** delivery system, interventional endoscopy, pigtail prothesis, pancreatic fluid collection, necrotizing pancreatitis

## Abstract

**Background and Objectives:**

EUS-guided drainage using lumen-apposing metal stents (LAMSs) has become standard treatment of symptomatic walled-off pancreatic necrosis (WOPN). Delivery systems with integrated electrocautery (EC) enable direct access and stent deployment, whereas the conventional stent insertion requires several steps including access using a needle or cystotome, wire insertion, and enlargement of the created tract before the stent placement. This study aimed to compare the practicality of EUS-guided procedures and their outcomes between conventional LAMSs (cLAMSs) and integrated EC (EC-LAMSs).

**Methods:**

In consecutive patients undergoing EUS-guided LAMS insertion with or without integrated EC, data on procedure time, sedation, and technical and clinical success, as well as adverse events, were analyzed.

**Results:**

From our prospectively maintained database, we analyzed 107 consecutive procedures of transmural EUS-stenting for drainage of WOPN. Thirty-nine cLAMSs and 68 EC-LAMSs were placed. Mean WOPN size was 12.9 ± 4.2 cm with mean 30% ± 15% solid necrosis.

Procedure times were shorter with EC-LAMSs (18.0 ± 6.6 *vs.* 39.7 ± 8.3 minutes; *P* < 0.05). All EUS-guided procedures with cLAMS drainage were performed under propofol sedation administered by anesthetist. In the EC-LAMS group, 36 patients tolerated the procedure under intravenous sedation (fentanyl/midazolam), and 17 interventions were performed as day case procedures. Adverse events and clinical outcome did not differ between both groups.

**Conclusion:**

Compared with cLAMS, EC-LAMS deployment is faster and technically less demanding. This allows performing the procedure under conventional intravenous sedation and as day case procedure in selected patients.

## Introduction

EUS-guided transmural stenting has become the standard treatment for patients with symptomatic walled-off pancreatic necrosis (WOPN) resulting from necrotizing pancreatitis.^[1–4]^ The development of short-length lumen-apposing metal stents (LAMSs), fully covered self-expanding metal stents with a special design of the flanges and large diameter, improved the drainage of debris and facilitated further direct endoscopic necrosectomy.^[5,6]^ In 2012, Itoi et al. published the first report of EUS-guided drainage of peripancreatic fluid collections using LAMSs.^[7]^ When correctly positioned, the specific design of the fully covered LAMSs, the dumbbell shape and radial expanding forces, keeps the stent in place and reduces the risk of migration and leakage of pancreatic or intestinal fluids.

Due to these advantages, the use of LAMSs for the treatment of walled-off necrosis (WON) has significantly increased in the last decade as it offers a minimally invasive approach. Although serious immediate or delayed adverse events are rare, the EUS-guided placement of LAMSs can become complicated by bleeding, infection, occlusion, perforation, stent maldeployment, or migration. Overgrowth of the stent with hypertrophic gastrointestinal mucosa (“buried stent”) is a rare adverse event in long-term indwelling stents.

The development of delivery systems with integrated electrocautery (EC) has simplified the placement of the LAMSs as they enable direct access to the necrotic cavity and stent deployment without exchange of instruments over the wire. The conventional method of stent insertion requires several steps including access to the WON using a needle or cystotome, followed by wire insertion and enlargement of the created tract before the stent placement can take place. There is a risk of leakage and/or losing position and access to the cavity with these multiple steps.

The EC-enhanced LAMSs (EC-LAMSs) combines access, tract widening, and stent deployment in one device and allows a single operator procedure.

In this prospective study, we aimed to compare the practicality of EUS-guided procedures and their outcome between conventional LAMSs (cLAMSs) and those with integrated EC (EC-LAMSs).

## Methods

### Study design

From our prospectively maintained database, consecutive patients with WOPN who were referred for EUS-guided drainage after discussion at the benign hepatopancreaticobiliary multidisciplinary team meeting were included. WOPN was reported according to the revised Atlanta classification^[8]^ as an organized collection with both solid necrotic and liquid material developing approximately 4 weeks after the onset of acute pancreatitis symptoms. Patients with pancreatic pseudocysts were excluded.

Infection of the WOPN, biliary compression, gastric outlet obstruction, and persistent pain after 6 weeks due to a large collection were accepted indications for intervention. The time of intervention was at least 4 weeks after onset of symptoms of acute pancreatitis.

Data access and analysis were approved by the appropriate institutional authorization (Caldicott approval). All aspects of the study were conducted in accordance with the Declaration of Helsinki 1964, as revised in Tokyo 2004. Written informed consent was obtained from all patients prior to the procedure.

### Data collection

#### Definitions

Technical success was considered as the deployment of the LAMSs into the correct position with the distal flange in the WOPN and the proximal flange in the gastrointestinal lumen.

Clinical success was defined as reduction of the collection to less than 4 cm and resolution of symptoms at 6 months’ follow-up.

The procedure time was defined as time when the intravenous sedation was administered to the time when the endoscope was withdrawn. The time points were taken from the endoscopy nursing documentation and/or anesthetic chart.

Adverse events were recorded and graded according to the American Society of Gastroenterology lexicon.^[9]^

#### EUS intervention

EUS-guided drainage of WON was performed in left lateral position under intravenous sedation with fentanyl/midazolam administered by the gastroenterologists or propofol sedation given by an anesthetic team. In patients with endotracheal intubation, the EUS was performed in supine position under propofol sedation.

For EUS intervention, therapeutic linear echoscopes with a 3.8 -mm instrument channel (EG-3870UTK; Pentax, Tokyo, Japan) and Arietta V70 platform were used (Hitachi Medical Corp., Tokyo, Japan).

Two endoscopists experienced in interventional EUS with more than 5 years of experience performed the procedure. The maximal diameter of the necrotic collection was measured endosonographically before attempting drainage. The endoscopists estimated the percentage of solid material in the WOPN from the EUS images before starting the drainage procedure.

After excluding vessels in the intended tract by color Doppler sonography, access to the WOPN was obtained using a 19-gauge needle or cystotome (Cook Medical, Winston-Salem, NC) in case of cLAMSs (NAGI [Taewoong Medical, Gyeonggi-do, South Korea] or Hanaro [M.I.Tech, Gyeonggi-do, South Korea]) and via direct diathermy of the integrated EC system when using the EC-LAMSs (Hot AXIOS; Boston Scientific, Marlborough, MA).

All patients received intravenous antibiotics during the procedure, and an antibiotic course was continued for at least 3 days postprocedure.

Day case patients were observed for 4 hours and clinically assessed before discharge by the endoscopists. Patients were informed about potential complications such as fever, bleeding, and pain and were given a contact number to seek immediate advice as needed.

Unless needed earlier for clinical needs, a computed tomography of the abdomen was scheduled 4 weeks after the procedure. If the collection was less than 4 cm in the follow-up scan, the LAMSs were extracted. The stent was left in place for further 4 weeks if the WOPN was still more than 4 cm until further imaging again in 4 weeks. Follow-up appointment was arranged in the outpatient department

### Statistical analysis

Primary endpoints were the technical and clinical success. Secondary endpoints comprised the occurrence of adverse events, sedation requirements, hospital stay, and procedure times.

Continuous variables are reported as mean and SD or median and range as appropriate. Missing data were excluded, and percentages were based on the number of nonmissing values. Categorical variables are presented as proportion with 95% confidence interval. Fisher *t* test was used. *P* < 0.05 was considered statistically significant.

## Results

From January 2017 to June 2023, 113 patients with WOPN were referred for EUS-guided drainage. Mean age was 55 ± 14 years. Most of the patients were male (65%). Mean size of the WON was 12.9 ± 4.2 cm with mean 30% ± 15% solid necrosis as estimated by the endoscopist performing the EUS.

**Figure 1:**
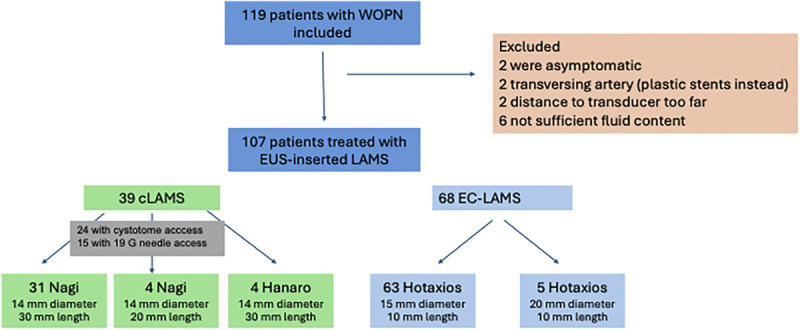
Flowchart of patients in this study.

Two patients were excluded as they had become asymptomatic at the time of the procedure. In 2 other patients, the procedure was not performed using a LAMS as a transversing artery was visible in the WON, and instead plastic stents were inserted. In 2 other patients, the distance of the WON to the transducer was considered too far during EUS, and percutaneous drainage was placed. In 6 patients, the EUS procedure could not be performed without fluoroscopy as the solid content of the WOPN was too high and hyperechogenic to allow endosonographic visualization of the stent placement steps. Figure 1 shows the flowchart of patients in this study.

Table 1 shows the baseline characteristics of the patients undergoing EUS-guided LAMS insertion in both groups.

**Table 1:** Baseline characteristics of patients undergoing EUS-guided transmural stenting for drainage of walled-off pancreatic necrosis using conventional lumen-apposing metal stents (cLAMSs) or electrocautery enhanced LAMSs (EC-LAMSs).

	cLAMSs *n* = 39	EC-LAMSs *n* = 68	*P*
Patients			ns
female/male	13 female/26 male	24 female/44 male	ns
Age	54 ± 17 yr	55 ± 13 yr	
Cause of pancreatitis	22 biliary	32 biliary	ns
	15 alcohol	24 alcohol	
	2 unclear	2 postsurgery	
		2 drugs	
		4 unclear	
		4 others	
Leading symptoms*	27 gastric outlet obstruction	39 gastric outlet obstruction	ns
	10 biliary compression	17 biliary compression	
	14 infected collection	21 infected collection	
	14 persistent pain	24 persistent pain	
Size of WOPN	14 ± 3 cm	12 ± 5 cm	ns
Solid debris %	32% ± 15%	28% ± 22%	ns

*Multiple symptoms in the same patient.

Technical success of correct stent placement was 97.4% in the cLAMS group *versus* 97.1% in the EC-LAMS group.

In one patient in the cLAMS group, the opening of the distal flange in the WOPN could not be visualized due to high percentage of hyperechogenic solid material in the collection. Instead, a pigtail stent was placed under fluoroscopy guidance. In the EC-LAMS group, with the distal flange in the WOPN, the proximal flange of one stent had been set free in the instrument channel, but then the endoscopist inadvertently pulled the entire stent out with the endoscope. In another patient, the stent was misplaced into the collection; directly thereafter, a second EC-LAMS was placed in correct position. Six days later, the misplaced stent could be successfully retrieved from the WOPN through the correctly placed stent after the critically ill and septic patient had been stabilized.

The clinical outcome after 6 months did not differ between both groups (Table 2). The mean follow-up was 28 ± 11 months.

**Table 2:** Outcome in patients undergoing EUS-guided transmural stenting for drainage of walled-off pancreatic necrosis using conventional lumen-apposing metal stents (cLAMSs) or electrocautery-enhanced LAMSs (EC-LAMSs).

Results	cLAMSs (*n* = 39)	EC-LAMSs (*n* = 68)	*P*
Access via			
19-Gauge needle	15 (38.5)	n/a	
Cystotome	24 (61.5)	n/a	
Technical success	38/39 (97.4%)	66/68 (97.1%)	1.00
Clinical success	31/39 (79.5%)	56/68 (82.4%)	0.79
Procedure time	40 ± 8 min	18.0 ± 7 min	**<0.001**
Sedation			
General anesthesia with			**<0.001**
Endotracheal intubation	36	10	
Intravenous propofol	3	17	
Intravenous fentanyl/midazolam	0	41 without anesthetists	
Day case procedure	0	17	**<0.001**
Endoscopic necrosectomy	8/39 (20.5%)	17/68 (25.0%)	0.64
Adverse events	6	9	0.78
Bleeding	2	5	
Perforation	0	0	
Stent dislocation	2	1	
Occlusion	2	2	
Buried bumper	0	1	
Death (not procedure related)	1	5	

*P*-values <0.05 indicate significant differences between both groups.

In the c-LAMS group, all stent insertions in patients with WOPN were performed with the support of an anesthetic team. In the EC-LAMS group, 41 patients (60.3%) tolerated the procedure under light sedation with fentanyl/midazolam. Both endoscopists rated the technical procedural challenge of the cLAMS placement higher than the insertion of EC-LAMSs.

## Discussion

Our retrospective observational study aimed to ascertain the practicality and outcomes of cLAMSs *versus* EC-LAMSs for the drainage of WOPN. Although the technical and clinical success in our study was not statistically significant between both stent types, EC-LAMSs outperformed cLAMSs in the simplicity of deployment and the time required for the procedure. Insertion of cLAMSs requires several procedural steps with exchange of tools over the guidewire, which takes more time. Each step poses a risk of losing the position and access to the pancreatic cavity. After the access tract is dilated using a balloon catheter or the 10F cystotome diathermy probe, the tool is extracted while only the wire stays in place. During this time, before the self-expanding LAMSs is placed, leakage of fluid content from the WOPN is possible. This can result in retroperitoneal leakage when the WOPN is not fixated due to inflammatory processes to the gastric or duodenal wall.

Placing an EC-LAMS is technically less demanding. Guidewires are not needed, and there is no exchange of instruments. It is a single operator procedure; the handle of the deployment set consists of a distal portion for catheter control and a proximal portion for stent control. Therefore, the endoscopist can deploy the stent from the handle of the system without the need for a skilled nursing team.

Although the single-device stent placement using EC-LAMSs appears to be easier and more intuitive and straightforward than cLAMSs, it is extremely important to have the specific competences to deal with potential complications such as maldeployment, bleeding, and perforation. In situations of misplacement, the conventional Seldinger technique and access to the cavity over a wire might enable rescue options. Therefore, it should be mandatory that EUS endoscopists and the endoscopy team gain proficiency and competence also in the over-the-wire-exchange techniques through appropriate training in *ex vivo* models and in supervised *in vivo* procedures.

There is plenty of literature comparing pigtail stent insertion with EC-LAMSs, but studies directly comparing EC-LAMSs and cLAMSs are rare and usually not limited to WON.^[10]^

Previous studies also found shorter procedure times for the placement of LAMSs compared with pigtail prothesis insertion in the management of WOPN (35 *vs.* 45 minutes, *P* = 0.003).^[11]^ A meta-analysis reported a pooled difference of stent insertion times of 26 minutes in favor of LAMS implantation in WON (−26.16 minutes [95% confidence interval, −28.68, −23.65], *I*^2^ = 0%, and *P* < 0.00001).^[12]^ Compared with the insertion of long biflanged metal stents^[6]^ and long self-expanding metal stents,^[13]^ the placement of EC-LAMSs proved also to be significantly shorter.

In the EC-LAMS group, 17 patients could be discharged on the same day, whereas in the cLAMS group, none left the hospital the same day. As there was no difference in comorbidities or severity of acute pancreatitis between the groups, this might be explained by several factors: (*a*) during the deployment of the LAMSs without integrated EC, there are steps when leakage of pancreatic fluids could occur. Especially, during exchange of tools after widening the new access tract and before the covered self-expanding stent is placed sealing the opening to the sides, fluids might leak next to the guidewire. If there is no fixation to the gastric wall, such fluid leakage might cause pain. (*b*) Patients are usually kept overnight in our hospital after general anesthesia with endotracheal intubation. (*c*) Over the years, our threshold to discharge symptom-free patients after 4 hours of postprocedural clinical observation probably has lowered.

Comparing different studies in this field remains problematic due to the lack of consistent definitions of outcomes, severity of pancreatitis, procedure times, and adverse events. In addition, the heterogeneity of the fluid collections included, the degree of necrotic material in the collection, and the follow-up varies substantially. A strength of our study is that only patients with WON were included.

Limitation of our study is its nonrandomized nature. The data were retrieved from a prospectively maintained database including all consecutive transmural EUS interventions, but the selection of cLAMSs or EC-LAMSs has not been randomized. However, before March 2020, we used only cLAMSs, and thereafter only-EC-LAMS, thus selection bias is not given. However, growing expertise of the entire team of endoscopists, nurses, and anesthetists in this kind of procedure might have contributed to shorter procedure times. We did not perform a detailed cost-effectiveness analysis.

In conclusion, the findings of this study suggest that cLAMSs and EC-LAMSs achieve similar rates of technical and clinical success without difference in the rate of adverse events.

EC-LAMS deployment is faster and technically less demanding. This might allow performing the procedure under conventional intravenous sedation and as day case procedure in carefully selected patients.

## Acknowledgment

None.

## Source of Funding

None.

## Ethical Approval

Data access and analysis were approved by the appropriate institutional authorization (Caldicott approval). All aspects of the study were conducted in accordance with the Declaration of Helsinki 1964, as revised in Tokyo 2004.

## Informed Consent

Written informed consent was obtained from all patients.

## Conflicts of Interest

Drs Braden and Bekkali received a travel grant from Boston Scientific. Christoph F. Dietrich is a Co-Editor-in-Chief of the journal. The article was subjected to the standard procedures of the journal, with a review process independent of the editor and his research group.

## Author Contributions

Barbara Braden and Noor Bekkali performed the endoscopies. Barbara Braden designed the study and drafted the manuscript. All authors have contributed to this study and wrote the paper, and the final version has been read and approved by all.

## Data Availability Statement

The datasets generated and analyzed during the current study are not publicly available, but the anonymized data are available from the corresponding author on reasonable request.
